# Evaluation of the Effectiveness of Treatment with Dexamethasone Intravitreal Implant in Cystoid Macular Edema Secondary to Retinal Vein Occlusion

**DOI:** 10.1155/2018/3095961

**Published:** 2018-08-02

**Authors:** Simone Donati, Carlo Gandolfi, Simona Maria Caprani, Jennifer Cattaneo, Laura Premoli, Claudio Azzolini

**Affiliations:** Ophthalmology Clinic, Department of Medicine and Surgery, University of Insubria-ASST Sette Laghi, Varese, Italy

## Abstract

**Purpose:**

To evaluate retinal functional improvement by means of visual acuity and retinal sensibility examination after intravitreal dexamethasone implant in patients affected by cystoid macular edema secondary to retinal vein occlusion.

**Methods:**

Twenty-six consecutive patients affected by retinal vein occlusion complicated by cystoid macular edema were enrolled in this prospective interventional study. All patients underwent a baseline complete ophthalmological evaluation as well as retinal angiography, OCT examination, and microperimetry evaluation. Each patient was treated with intravitreal injection of a long-term steroid implant (Ozurdex, Allergan). Follow-up evaluations were performed at months 1, 3, and 6 and completed by OCT and MP1 examination. Clinical data underwent statistical analysis.

**Results:**

Baseline functional evaluation showed mean visual acuity of 0,63±0,42 LogMAR and retinal sensitivity of 7,93±4,73 dB (mean±standard deviation); after treatment, at day 30 we found, respectively, 0,43±0,38 LogMAR (p<0.05, compared to baseline) and 10,15±4,410 dB (p<0.05); at day 90, we found 0,44±0,32 (p<0.05) and 9.61±4,29 dB (p<0.05); at day 180, we found 0,41±0,31 (p<0.05) and 9,95±3,79 dB (p<0.05). Fixation pattern improved significantly (p<0.05), showing a stable fixation in 30% of patients at baseline, increasing to 77% of patients at day 180. Baseline morphological evaluation showed a central retinal thickness (CRT) of 398,21±181,65 *μ*m after treatment; we found a CRT of 222,64±95,21 *μ*m at day 30 (p<0.05, compared to baseline), 307,50±120,25 *μ*m (p<0.05) at day 90, and 294,93±135,86 *μ*m (p<0.05) at day 180. About 15,3% patients showed already at month 3 a recurrence of macular edema. They underwent a retreatment before month 6 as for treatment guidelines.

**Conclusion:**

Our detailed analysis showed the significative increase in retinal function in the early phases of the follow-up. Retinal sensibility showed a stronger correlation than VA in macular edema reabsorption, better underlying the progressive functional recovery and increase in quality of vision and life for the patients. This trial is registered with ClinicalTrials.gov NCT03559491.

## 1. Introduction

Cystoid macular edema represents the most important cause of visual impairment after retinal vein occlusion. The etiological factors of this complication are under investigation, from inflammatory cytokines to the breakdown of vascular barrier with increased permeability and diffusion of angiogenic factors [1, 2]. Clinical research developed different drugs and therapeutic strategies to treat and control macular edema, to evaluate the correct timetable, and to reach long-term clinical significative results. Now, different experiences have been published with available drugs, from steroids to anti-VEGF [3–7]. A debate still remains about the correct approach, the correct combination of drugs (if necessary), and the correct evaluation of drug effectiveness [8].

In our study, we evaluated extensive functional recovery and its relation to macular thickness after steroid injection. Considering that visual acuity reflects only foveal function, it may not be sufficient to evaluate the overall function on the macular area. We introduced microperimetry study as a valuable and reproducible tool to create a functional mapping of the entire macula.

## 2. Materials and Methods

This prospective, interventional noncontrolled study was conducted between July 2015 and August 2017 at the Medical Retina Service, Ophthalmology Clinic, Department of Medicine and Surgery, University of Insubria and ASST-Sette Laghi in Varese, Italy.

Patients affected by cystoid macular edema (CME) due to retinal vein occlusion of recent onset (less than three months) were enrolled. Inclusion criteria were as follows: age older than 18 years; CME secondary to retinal vein occlusion; disease duration inferior to 12 weeks; best-corrected visual acuity (BCVA) between 20/200 and 20/25 (Snellen equivalent) in the study eye at baseline examination and central retinal thickness (CRT) superior to 300 mm, as measured by SD-OCT at baseline examination.

The exclusion criteria were any ocular surgery in the study eye in the past 6 months; diabetes mellitus with signs of diabetic retinopathy; previous laser photocoagulation; previous intravitreal injection of corticosteroids or antivascular endothelial growth factor; a history of ocular inflammation; marked retinal ischemia or large retinal hemorrhages (in particular in macular region involving the fovea); any other ocular condition such as anterior ischemic neuropathy, amblyopia, or significant media opacities; significant alteration on epiretinal surface such as epiretinal membrane or vitreomacular traction; uncontrolled or advanced glaucoma; any uncontrolled systemic disease.

The study was conducted in accordance with the ethical standards of the Declaration of Helsinki. Informed consent was obtained from all patients before the inclusion in the study as the standard as approved care for this pathology. This clinical study was approved by the ethical committee board of our hospital, as the application of a standard of care for this pathology.

At baseline all patients were treated with intravitreal injection of dexamethasone 0,7 mg implant (Ozurdex; Allergan, Irvine, CA, USA). This implant was injected directly into the vitreous through sclerocorneal limbus at pars plana (3.5-4 mm) by means of a customized single use 25G injector. Intravitreal injection was performed in a surgical room, according to Italian Society of Ophthalmology guidelines, using a sterile kit comprehending sterile drape, gloves, and eyelid speculum. A complete disinfection was made before the injection with povidone iodine 5% on a sterile bottle, followed by topical anesthesia with oxybuprocaine chloride drops.

A pretreatment prophylaxis was made 3 days before and after the injection with broad-spectrum antibiotics topical drops.

A day one and day five posttreatment visit was performed to evaluate adverse events after the treatment.

At baseline a complete ophthalmological examination was performed, including ETDRS visual acuity, Goldmann applanation tonometry, and clinical evaluation of the fundus.

Instrumental evaluation included OCT examination, fluorescein angiography, and microperimetry.

OCT examination was performed by means of SD-OCT (Topcon 3DOCT, Tokyo, Japan). We performed topography map to evaluate central retinal thickness (CRT), two vertical and horizontal high resolution B-scans to study the microstructure of the retina, and the choroidal thickness evaluation (CT). CT was evaluated by means of a manual caliper in the foveal region (FCT), at 500 micron nasally and temporally to the fovea.

Fluorescein angiography was performed with HRA 2 (Heidelberg Engineering, Heidelberg, Germany).

The evaluation of retinal sensitivity was performed by means of MP-1 microperimetry (Nidek Technologies, Padova, Italy). The MP-1 performs microperimetry with an automated eye tracking system, which provides real-time compensation for eye movements and allows improved presentation of a stimulus at the predefined retinal location. The retinal sensitivity can be measured easily because the level of stimulation changes automatically and progressively during microperimetry. To generate retinal sensitivity maps, customized grid of 45 Goldmann III stimuli, covering the central 12° (centered on the fovea), were presented in random order according to a 4-2-1 double-staircase strategy. The stimulus intensity ranged from 0 dB to 20 dB (0 dB corresponded to the strongest signal intensity of 127 cd/m2) in 1 dB steps, and the duration of each stimulus was 200 milliseconds. The fixation target was varied in size according to the patient's visual acuity.

**Figure 1:**
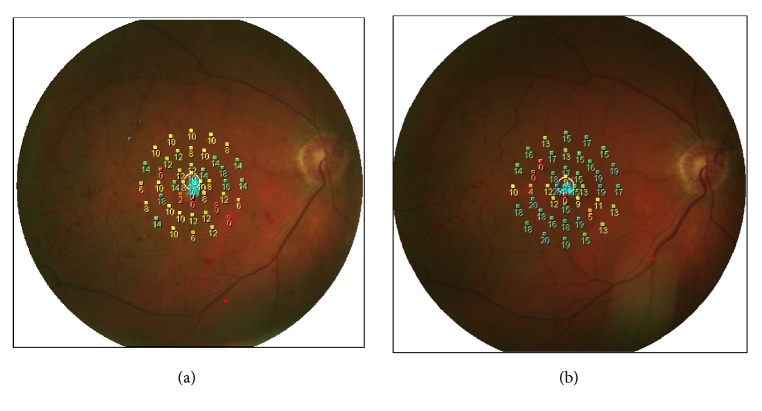
Microperimetry evaluation at baseline and at six months in a patient with CRVO.

Follow-up visits were performed at months 1, 3, and 6 including visual acuity measurement, complete ophthalmological evaluation, OCT examination, and microperimetry (Figure 1).

To consider a retreatment or a safe treatment, we consider as recurrence of macular edema an increase of CRT of more than 150 microns at month 3 compared to baseline. We consider as nonresponders to the treatment patients showing an increase of CRT of more than 150 microns at 1 month from the injection. In this case the patient was excluded from the study.

### 2.1. Outcome Measures

We considered as primary outcomes mean change in visual acuity, central retinal thickness, and retinal sensitivity, all measured in follow-up visits compared to baseline; we considered as secondary outcomes choroidal thickness and its variation during follow-up as well as the correlation between functional and morphological parameters. The measurement of incidence of side effects after intravitreal Ozurdex injections was considered a safety outcome.

### 2.2. Statistical Analysis

Collected data were analyzed by means of paired T test. To evaluate correlation between different categories of continuous data, a Pearson data test was applied. A statistical value of p≤0.05 was considered for statistical significance.

## 3. Results

Twenty-six patients (12 males, 14 females, mean age 67±5 years; range 56-81) were enrolled in the study and completed the follow-up. According to the diagnosed pathology, 18 patients were affected by nonischemic central retinal vein occlusion and 8 patients were affected by nonischemic branch retinal vein occlusion involving macular region. Mean duration of the pathology before treatment was 4.5 weeks and all patients, as for inclusion criteria, were naive.

**Figure 2:**
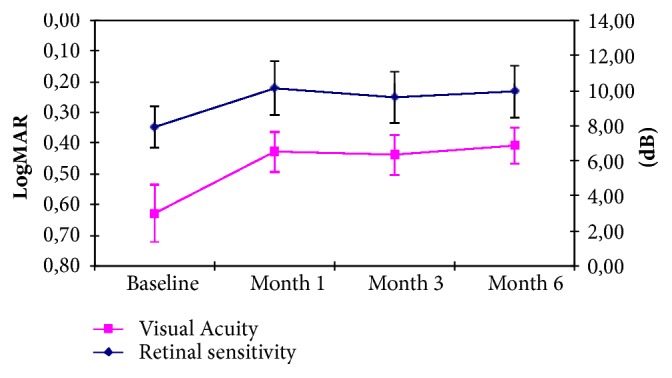
Mean visual acuity and retinal sensitivity variation during follow-up.

Baseline functional evaluation showed mean visual acuity of 0,63±0,42 LogMAR and retinal sensitivity of 7,93±4,73 dB (mean±standard deviation); after treatment, at day 30 we found, respectively, 0,43±0,38 LogMAR (p<0.05, compared to baseline) and 10,15±4,410 dB (p<0.05); at day 90, we found 0,44±0,32 (p<0.05) and 9.61±4,29 dB (p<0.05); at day 180, we found 0,41±0,31 (p<0.05) and 9,95±3,79 dB (p<0.05) (Figure 2).

Fixation pattern improved significantly (p<0.05), showing a stable fixation in 8 of 26 patients (30%) at baseline, in 16 patients (61%) at day 30, in 14 patients (53%) at day 90, and in 20 patients (77%) at day 180.

**Figure 3:**
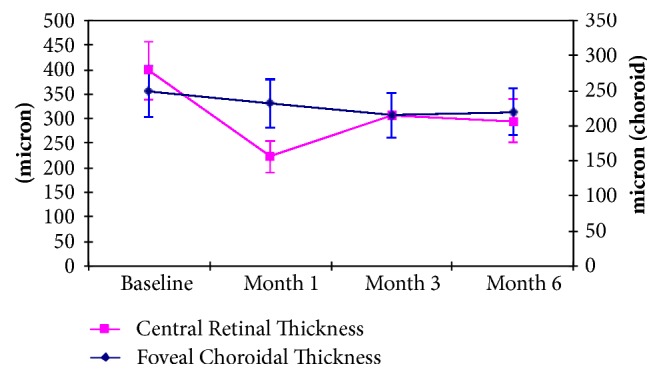
Mean CRT and mean FCT variation during follow-up.

Baseline morphological quantitative evaluation showed a central retinal thickness of 398,21±181,65 *μ*m; after treatment, we found a CRT of 222,64±95,21 *μ*m at day 30 (p<0.05, compared to baseline), 307,50±120,25 *μ*m (p<0.05) at day 90, and 294,93±135,86 *μ*m (p<0.05) at day 180 (Figure 3). Six patients (23%) presented at baseline a subfoveal neurosensory retinal detachment as complication of severe cystoid macular edema. This clinical feature resolved after therapy and the retina appeared adherent with a significative reduction of macular edema at month 6 in responsive patients.

A limited proportion of patients, 15,3% (4 out of 26 patients), already at month 3, showed a recurrence of macular edema. They underwent a retreatment before month 6 as for treatment guidelines.

No significant adverse events were recorded. A slight increase of intraocular pressure was evident in 4 patients at month 1 after treatment. After topical therapy with beta-blockers drops, the intraocular pressure, at months 3 and 6, was recorded within normal values.

All patients were phakic: we reported a nonsignificant increase of lens opacity in all patients; this element did not represent a bias in this study for the functional evaluation or for OCT study.

No significative inflammatory reactions were reported after the injection, as well as pain or ocular burning in any patient.

## 4. Discussion

Our study showed a significant increase in visual functions since the earlier follow-up period. In particular visual acuity and retinal sensitivity increased during the first month with stabilization on the following months (Figure 2). We know that microperimetry study is able to investigate retinal function in overall macular region. As well demonstrated for other macular pathologies like diabetic retinopathy, retinal sensitivity well correlates with function ability of the patients, investigating the fixation capability and evaluating the center of fixation, often modified in patients with severe macular edema [9–12]. Microperimetry allows a point-to-point examination, with a tracking system to maintain the fixation and the related studied region.

Considering retinal morphological characteristics, our study showed the long-term efficacy of steroid implant to control macular edema. OCT analysis showed a significant progressive reduction of CRT in all visits (Figure 3). We described the reduction of intraretinal cysts and the reabsorption of subretinal fluid in patients with subfoveal retinal detachment [13, 14]. The duration effect appeared otherwise inferior compared to the RCTs, as demonstrated in previous studies [7, 15, 16]: the need for a retreatment appeared less than 6 months, in order to ensure an effective control of macular edema recurrences and the visual acuity preservation in all patients. As described above, only 4 patients showed a recurrence of significant macular edema and were retreated earlier [8, 17–19]. Figure 3 shows at the same time a modification of foveal choroidal thickness, a discussed parameter. The clinical significance of this modification is disputable: a reduction of uveal vascular congestion or the organization of retinal vein drainage could modify choroidal vascular pressure.

**Figure 4:**
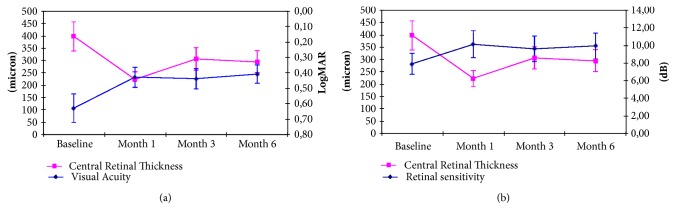
Mean CRT variation, respectively, compared to mean BCVA (a) and mean RS (b) variation during follow-up.

The analysis of correlation between functional and morphological improvement showed interesting data (Figures 4(a) and 4(b)). Macular sensitivity improvement appeared significantly correlated with visual acuity improvement (r:-0.9; p<0.05); this observation is important and it has been previously demonstrated by Vujosevic et al. [11] in patients treated for diabetic macular edema. Central fixation increased from 30% to 77% of our patients as well as its stability, from 45% to 90%.

**Figure 5:**
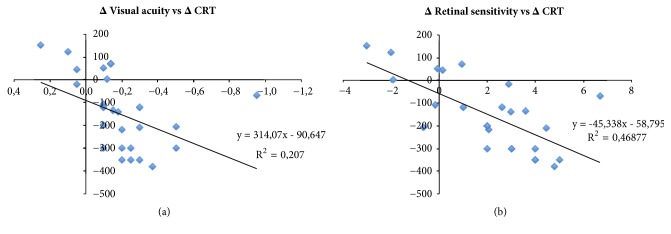
Correlation between mean CRT variation, respectively, compared to mean BCVA (a) and mean RS variation (b) at month 6.

Moreover we compared macular sensitivity, visual acuity, and central retinal thickness variations at month 6. We found interesting correlation between these data (Figures 5(a) and 5(b)). In particular, the microperimetry analysis appeared to better detect functional improvement related to macular edema reduction than visual acuity (r: 0,68 versus r: 0,45; p<0.05).

These data confirm the importance of a deep analysis of visual function in patients affected by macular edema [17, 20]. The presence of intraretinal cysts, intraretinal layers disruption, hemorrhages, and foveal depression loss could modify macular sensitivity and the possibility of fixation, affecting in this way the quality of central visual function. Microperimetry examination, evaluating the overall macular function, could get over these lesions and give to the examiner a real perception of functional recovery [13, 14, 17–19].

The strength of our study has been the possibility of evaluating the relation between different functional parameters during morphological recovery in this important vascular disease.

We identified as well some limits of the study, in particular the heterogeneity of the sample and the number of subjects. We decided to include the central and the branch retinal vein occlusion patients as they present in both cases a significant cystoid macular edema with the same characteristics and behavior. We identified a possible bias represented by the learning effect of patients during multiple microperimetry evaluations, well known event that we tried to reduce by a preliminary adaptive test before microperimetry examination.

## 5. Conclusion

In conclusion, our detailed analysis showed the significative increase in retinal function in the early phases of the follow-up. Intravitreal steroid is able to reduce macular edema and to recover visual functions since the first months of follow-up. Retinal sensibility showed a stronger correlation than VA with macular edema reabsorption, underlying better progressive functional recovery and increase in quality of vision and life for the patients.

## Data Availability

Clinical data used to support the findings of this study are included within the article.
